# Poverty Status at Birth Predicts Epigenetic Changes at Age 15

**DOI:** 10.31586/jbls.2024.989

**Published:** 2024-07-16

**Authors:** Shervin Assari, Hossein Zare

**Affiliations:** 1 Department of Internal Medicine, Charles R. Drew University of Medicine and Science, Los Angeles, CA, United States; 2 Department of Family Medicine, Charles R. Drew University of Medicine and Science, Los Angeles, CA, United States; 3 Department of Urban Public Health, Charles R. Drew University of Medicine and Science, Los Angeles, CA, United States; 4 Marginalization-Related Diminished Returns (MDRs) Center, Los Angeles, CA, United States; 5 Department of Health Policy and Management, Johns Hopkins Bloomberg School of Public Health, Baltimore, MD, United States; 6 School of Business, University of Maryland Global Campus (UMGC), College Park, MD, United States

**Keywords:** Epigenetic aging, DNA Methylation, Socioeconomic Status, Race, Ethnicity, Fragile Families and Child Wellbeing Study, PhenoAge, Health Disparities

## Abstract

Epigenetic studies have provided new opportunities to better understand the biological effects of poverty and racial/ethnic minority status. However, little is known about sex differences in these processes.

**Methods:**

We used 15 years of follow up of 854 racially and ethnically diverse birth cohort who were followed from birth to age 15. Structural equation modeling (SEM) was used to examine the effects of race/ethnicity, maternal education, and family structure on poverty at birth, as well as the effects of poverty at birth on epigenetic changes at age 15. We also explored variations by sex.

**Results:**

Our findings indicate that Black and Latino families had lower maternal education and married family structure which in turn predicted poverty at birth. Poverty at birth then was predictive of epigenetic changes 15 years later when the index child was 15. This suggested that poverty at birth partially mediates the effects of race/ethnicity, maternal education, and family structure on epigenetic changes of youth at age 15. There was an effect of poverty status at birth on DNA methylation of male but not female youth at age 15. Thus, poverty at birth may have a more salient effect on long term epigenetic changes of male than female youth.

**Conclusions:**

Further studies are needed to understand the mechanisms underlying the observed sex differences in the effects of poverty as a mechanism that connects race/ethnicity, maternal education, and family structure to epigenetic changes later in life.

## 1. Background

Epigenetic studies have introduced new tools to better understand biological influences of adversity [1-5]. Epigenetic clocks such as PhenoAge (phenotypic age) measure epigenetic aging based on DNA methylation (DNAm) levels at multiple cytosine-phosphate-guanine dinucleotides (CpGs) across the genome [6]. These tools are recently utilized to document biomarkers of aging, which is also under the influence of poverty [7]. Epigenetic age acceleration (AA) defined as older epigenetic age relative to chronological age is predictive of age-related diseases [6].

Epigenetic clocks can estimate epigenetic changes in various tissues such as blood or saliva [8]. Over the past decade, several advances have been made available epigenetic clock algorithms used in research [9]. The first-generation epigenetic clocks, including HorvathAge and HannumAge, predict chronological age using specific CpG sites strongly associated with chronological age [10]. Second-generation clocks, such as PhenoAge incorporate additional age-related clinical markers [11, 12]. The residual from regressing epigenetic age on chronological age is referred to as epigenetic age acceleration (AA) [13]. There are, however, newer generations of epigenetic clocks [14] that incorporate longitudinal within-person physiological changes along with methylation patterns, specifically designed to measure the rate of aging [14].

Second-generation clocks outperform first-generation clocks in predicting the risk of age-related diseases and all-cause mortality [15, 16]. Using the third-generation clock, DunedinPACE, a recent study documented the positive effects of high levels of education on the pace of aging, showing benefits irrespective of individuals’ genetics. The study used data from five studies across the lifespan and showed that high educational attainment is associated with a slower pace of aging even after accounting for genetic factors. This protective effect also persisted after controlling for tobacco smoking [17].

The effects of adversities, socioeconomic position (SEP), and race/ethnicity as sociological markers on biological aging, DNA methylation, and epigenetics are profound and interconnected [18]. Adversities such as chronic stress, early-life adversity, and socioeconomic disadvantage have been linked to accelerated biological aging processes, reflected in altered DNA methylation patterns [19-21]. These changes can influence gene expression and physiological functions, potentially predisposing individuals to various age-related diseases [22-24]. SEP plays a critical role as well, with lower socioeconomic status often associated with poorer health outcomes and accelerated biological aging [25]. Race and ethnicity, as social rather than biological factors, also intersect with adversities and SEP, through differential access to resources, exposures to environmental stressors, and societal discrimination, all of which can impact epigenetic mechanisms and biological aging trajectories [26-28]. Understanding these complex interactions is crucial for elucidating disparities in health outcomes across different population groups and for developing targeted interventions to mitigate the effects of socioeconomic and racial inequalities on aging and health.

## 2. Objective

This study explores the associations between race/ethnicity, maternal education, family structure, poverty at birth, and epigenetic changes at age 15. To be more specific, we tested whether poverty at birth partially mediates the link between racial/ethnic minority status and epigenetic aging at age 15.

## 3. Methods

### 3.1. Design and Setting

The Future of Families and Child Wellbeing Study (FFCWS), formerly known as the Fragile Families and Child Wellbeing Study, is a pioneering research project aimed at understanding the challenges faced by economically disadvantaged families in the United States. The FFCWS follows a birth cohort starting in 1998, tracking children from birth to young adulthood at age 22 in 2022. Detailed information on the study’s sampling techniques and methodologies can be found in previously published literature. This section provides a brief overview of the FFCWS research design. In the current analysis, we only used the first 15 rather than 22 years of the data.

### 3.2. Ethics

The study protocol was approved by the Institutional Review Board at Princeton University. Informed consent was obtained from all participating families, with parents or legal guardians consenting on behalf of minors, who also provided their assent. All data collection, storage, and analysis procedures were designed to protect participants' anonymity, and families were compensated for their participation.

### 3.3. Sample and Sampling

The FFCWS recruited a diverse sample of urban families from 20 major U.S. cities, each with a population exceeding 200,000. The study specifically targeted underrepresented families, particularly non-married, Black, and Latino families. Consequently, the study's sample predominantly consists of low socioeconomic status families, with a substantial representation of Black and Hispanic participants, which does not reflect the overall U.S. population. The analytical sample included 854 families with a Black, Latino, or White families. We selected the sample based on availability of epigenetic data at age 15.

### 3.4. Process

Our analysis utilized data from the first and seventh waves of the FFCWS. Socioeconomic position (SEP) data were collected at birth (wave 1, baseline), and outcomes were measured when the offspring were young adults 22 years later (wave 7). The analysis included 854 Black and White families with follow-up data.

### 3.5. Predictors

Baseline data were collected through interviews with both parents, covering parents' poverty status and family structure at birth. Family poverty status at birth was measured as a continuous measure ranging from 0 to 12.3, with higher number indicator of higher SEP.

### 3.6. Collection of Saliva Sample

During the Year 15 follow-up wave, saliva was collected from the focal children (now teenagers) using Oragene DNA Self-Collection Kits (OGR-500) as described for the year 9 followup with the following modifications. For those who did not complete a home visit, saliva collection kits were sent to participants via mail and after collection participants returned the kits to Westat via FedEx. Participants were discouraged from eating or drinking anything within 30 minutes prior to sample collection. Upon completion of the saliva collection, all participants received $20 [29].

### 3.7. Acquisition and Processing of the DNA Methylation Data

For the DNA methylation analysis, approximately 500 ng of genomic DNA, quantified using the Quant-iT Picogreen dsDNA Assay Kit, underwent bisulfite conversion utilizing the EZ-96 DNA Methylation Kit (Zymo Research). The converted DNA was then analyzed using the Illumina Infinium Human Methylation450K (450K) or the Illumina Infinium MethylationEPIC (EPIC) array, following the manufacturer's protocols. This process was carried out by the Pennsylvania State College of Medicine Genome Sciences Core facility. To minimize technical variation, DNA samples from ages 9 and 15 were processed concurrently. Samples were randomized to prevent bias. The red and green image pairs were imported into R for analysis. Quality control (QC) of the methylation data was conducted initially using EWAStools. Probes were excluded if their detection values exceeded 0.01 for the 450K array or 0.05 for the EPIC array, or if the number of methylated or unmethylated bead counts was less than four. Probes were also removed based on the ENmix function QCinfo, which was applied with default parameters. Samples were excluded if they had outlier methylation or bisulfite conversion values as identified by the ENmix QC function, or if the predicted sex from the methylation data did not match the recorded sex. Additionally, samples were flagged if sequential samples from the same individual showed genetic discordance between visits. The ENmix preprocessENmix and rcp functions were employed to normalize dye bias, apply background correction, and adjust for probe-type bias [29].

### 3.8. PhenoAge Epigenetic Clock

Developed by Levine, Horvath and colleagues, this epigenetic clock was trained using a two-step method to generate a lifespan predictor. The first step was a Cox penalized regression model in which the hazard of mortality was regressed on clinical markers (albumin, creatinine, serum glucose, C-reactive protein, lymphocyte percent, mean cell volume, red blood cell distribution width, alkaline phosphatase, and white blood cell count), and chronological age to predict phenotypic age. Elastic net regression where the phenotypic age was predicted by blood DNA methylation data was then used to identify the 513 CpGs comprising the DNAm PhenoAge measure [29].

### 3.9. Statistical Analysis

Data analysis was conducted using Stata version 18.0. Descriptive statistics, including frequencies (percentages) and means (standard deviations), were reported. Bivariate analysis was performed using the Pearson correlation test. For the multivariable analysis, we applied structural equation modeling (SEM) to examine the associations between race/ethnicity, poverty status at birth, and youth PhenoAge (the outcome) 15 years later. Structural Equation Modeling (SEM) is a powerful statistical technique that allows researchers to examine complex relationships between variables. It goes beyond traditional regression models by simultaneously analyzing multiple dependent and independent variables within a single framework. One of SEM's key advantages is its ability to test mediation and moderation effects. By incorporating latent variables (unobserved constructs) and observed variables into a unified model, SEM can assess how indirect relationships (mediation) and interactions (moderation) among variables contribute to outcomes of interest. This holistic approach not only enhances understanding of causal pathways but also provides a more nuanced perspective on how different factors interrelate in explaining phenomena. Therefore, SEM is invaluable in social sciences, psychology, economics, and other fields where understanding complex relationships is essential for theory development and practical applications. The analysis explored the impact of race/ethnicity on biological aging of the child at age 15 via poverty status at birth. We also controlled for family structure, sex, and ethnicity.

## 4. Results

### 4.1. Descriptive Statistics

Overall, 854 participants entered our analysis. Table 1 shows their descriptive data. 45.32% were Black, 19.32% were Latino, 23.42% were living in married households at baseline, and 50.12% were male.

### 4.2. Unadjusted Bivariate Correlations

Bivariate unadjusted correlations are shown in Table 2. As this table shows, PhenoAge (methylation) was inversely associated with higher SEP and being in a married family. At the same time, Black race was associated with higher methylation in youth. Male sex was also associated with higher PhenoAge (DNA methylation).

### 4.3. Adjusted Multivariable Results

**Figure 1.**
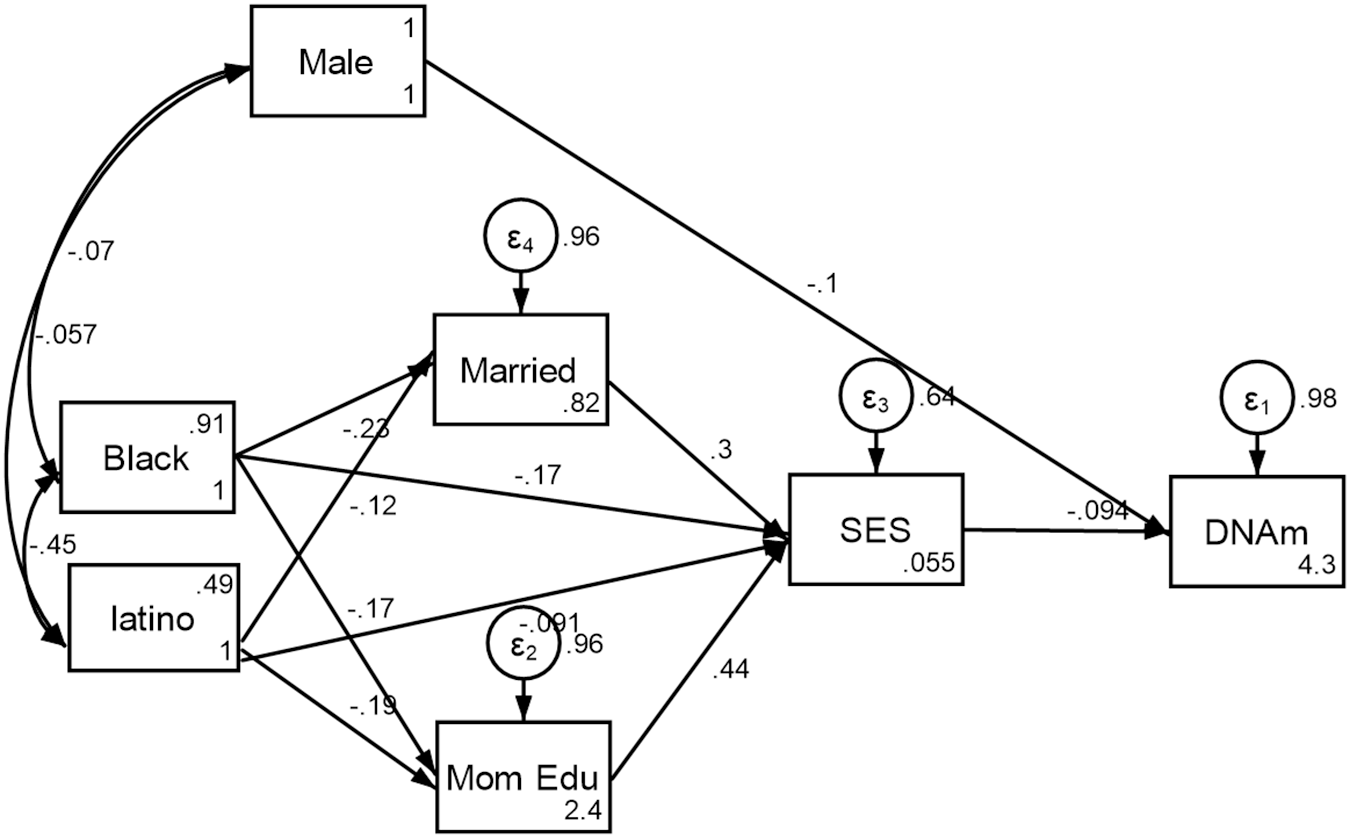
Summary of Structural Equation Modeling (SEM), Overall

Our findings indicate that Black race and Latino ethnicity were associated with poverty status at birth as well as faster epigenetic aging. Specifically, poverty status at birth partially mediated the effects of race and ethnicity on accelerated aging by age 15 (Figure 1, Table 3).

**Figure 2.**
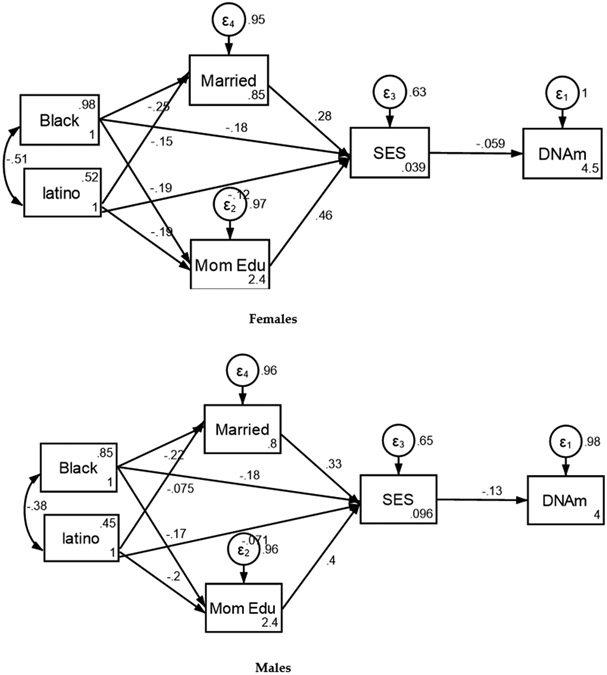
Summary of Structural Equation Modeling (SEM) by Sex

Our findings indicate that lower SEP at birth was a predictor of faster epigenetic aging of male not female participants in the sample. Specifically, higher poverty status at birth partially mediated the effects of race and ethnicity on accelerated aging by age 15 for males but not females (Figure 2, Table 4).

## 5. Discussion

Being Black and Latino is significantly associated with higher poverty, which is linked to accelerated epigenetic changes in male not female youth at age 15. Poverty at birth partially mediates the effect of race/ethnicity on DNA methylation of male not female youth. This suggests that while poverty at birth has a lasting impact on epigenetic changes into adolescence, for male and female Black and Latino youth.

We observed poverty has an effect on DNA methylation of male not female youth. The effects of SEP on health differs for males and females [30-32]. Sexualized racism and gendered racism highlight the complex interplay between race/ethnicity, gender, and sexuality, illustrating that men and women experience racism in distinctly different ways [33-35]. Sexualized racism refers to the unique discrimination faced by individuals based on both their race and perceived sexual characteristics or stereotypes. For example, women of color often endure hypersexualization and exoticization, leading to objectification and dehumanization in ways that White women do not experience. Conversely, gendered racism emphasizes how racial discrimination manifests differently for men and women. Black and Latino men, for instance, may encounter heightened scrutiny and criminalization, often being stereotyped as inherently violent or dangerous. On the other hand, Black women might face stereotypes of being overly aggressive or angry, while also navigating the intersection of sexism and racism in professional and social settings. These intersecting forms of discrimination underscore the necessity of considering both race/ethnicity and gender in understanding the full scope of racism and its varied impacts on different groups [36].

The effects of race and ethnicity on epigenetics changing can be explained by "weathering hypothesis". This hypothesis explains why Black individuals often exhibit signs of accelerated aging compared to their White counterparts [37, 38]. This hypothesis posits that the cumulative impact of social, economic, and environmental stressors—rooted in systemic racism—leads to premature biological aging in Black individuals. Geronimus' theory suggests that chronic exposure to adversity and discrimination experienced by Black communities results in "weathering," a term she uses to describe the wear and tear on the body due to prolonged stress [37, 38]. This concept draws an analogy to how environmental conditions can erode physical structures over time. In the context of human health, the persistent stress and strain from living in a racially stratified society can lead to earlier onset of chronic diseases, higher morbidity, and mortality rates. The weathering hypothesis is supported by substantial evidence showing that Black and Latino individuals often have higher levels of stress-related biomarkers, such as cortisol, and exhibit greater physiological dysregulation compared to Whites. This chronic stress leads to increased allostatic load, a measure of the cumulative biological burden exacted by chronic stress and life challenges. Geronimus' research has shown that these stressors not only affect mental health but also manifest physically, contributing to disparities in birth outcomes, cardiovascular health, and overall mortality [37-45]. The accelerated aging observed in Black and Latino individuals is not merely a result of genetics but is significantly influenced by the socio-economic and environmental context shaped by historical and contemporary racism. Her work underscores the importance of addressing the root causes of racial disparities in health by tackling the systemic inequities that perpetuate stress and disadvantage in Black and Latino communities. Interventions aimed at reducing these disparities must consider the broader socio-political landscape that influences health outcomes, emphasizing the need for policies that promote social justice and equity.

Racial/ethnic minority status is, in part, a proxy for exposure to racism and racialization. Black and Latino individuals are subjected to multi-level discrimination. Due to segregation, Black and Latino individuals have lower access to resources and buffers [46-51]. Moving out of poverty is less protective for Black and Latino families than for White families because of systemic racism. Chronic stress, often associated with minority status, influences the rate of aging. Financial instability, job insecurity, and social disadvantages increase stress, leading to dysregulation of the hypothalamic-pituitary-adrenal (HPA) axis [52], elevated cortisol levels, and increased allostatic load. Lower SEP affects dietary quality and nutrition. Families with limited access to healthy food options face poor nutrition, which can adversely affect metabolic processes and accelerate aging at the cellular level. Poverty is linked with higher exposure to environmental toxins, such as pollutants and lead. Impoverished neighborhoods often have closer proximity to industrial areas and substandard housing, increasing the risk of toxin exposure and inducing epigenetic changes that contribute to accelerated aging. Individuals from lower SEP backgrounds typically have reduced access to healthcare services, resulting in untreated health conditions and delayed diagnoses, which can exacerbate health issues and accelerate aging [53-56].

We see race and ethnicity as social rather than biological constructs [43, 57-59]. Racial and ethnic differences observed in health and aging metrics reflect the impact of historical, economic, societal, and environmental influences rather than innate biological distinctions [60-63]. These differences are the result of centuries of systemic inequalities, including discriminatory practices, socioeconomic disparities, and unequal access to resources and opportunities [64-66]. The lived experiences of marginalized racial and ethnic groups, shaped by persistent exposure to racism and segregation, contribute to the health disparities seen today [67, 68]. By understanding race/ethnicity as a social factor, we aim to highlight the profound effects of structural inequalities and advocate for systemic changes to address these disparities [62, 69-74].

Community-based interventions play a pivotal role in addressing systemic inequalities exacerbated by poverty and racism, particularly in their impact on health outcomes. These initiatives empower local residents and organizations to develop and implement tailored strategies that meet specific community needs and contexts. They encompass a range of services such as healthcare access, mental health support, nutritional programs, preventive care education, and advocacy for equitable policies. By directly engaging with affected communities, these interventions not only provide immediate support but also cultivate long-term resilience and capacity-building. Furthermore, they contribute significantly to breaking down structural barriers that perpetuate health disparities, thereby fostering a more inclusive and equitable society. Meaningful discussions and collaborations among policymakers, healthcare providers, and community leaders are essential to ensuring the effectiveness and sustainability of these interventions in tackling complex societal challenges.

Several policies have been implemented to alleviate the impact of poverty in marginalized communities by enhancing access to resources and opportunities. For example, targeted education initiatives such as early childhood education programs and scholarships for higher education aim to break the cycle of poverty by improving educational outcomes and increasing economic mobility. Affordable housing policies, including subsidized housing and rent control measures, provide stable living conditions and reduce homelessness risks. Additionally, policies promoting workforce development and job training programs equip individuals with skills necessary for sustainable employment, thereby boosting income levels and financial stability. Social welfare policies like food assistance programs (e.g., SNAP) and healthcare coverage expansions (e.g., Medicaid expansion) ensure basic needs are met, enhancing overall well-being and reducing health disparities. By addressing these diverse needs through comprehensive policy approaches, governments can effectively support marginalized communities in overcoming barriers to economic and social progress.

## 6. Implications

The findings of this study have significant policy and public health implications. Addressing socioeconomic inequalities at birth is crucial to mitigating the long-term biological impacts of racism on Black and Latino families. Policymakers should prioritize public, social, and economic interventions that reduce socioeconomic disparities and improve living conditions for disadvantaged families. Increasing access to quality education, healthcare, and nutritious food, along with reducing exposure to environmental toxins, could help slow down the pace of aging in marginalized communities. Poverty elimination is a major element of addressing racial disparities in accelerated aging. Increasing minimum wage and earned income tax benefits are among the vehicles by which poverty can be reduced in Black and Latino families.

## 7. Limitations

Several limitations should be considered when interpreting these findings. The study relies on observational data with considerable missing data, which cannot establish causality. Our measure of epigenetic aging was limited to PhenoAge, which may require further validation in diverse populations. While the study included poverty, maternal education, and family structure as SEP indicators, several unmeasured confounders could have influenced the observed associations. Long-term follow-up studies have high attrition that may cause bias. Wealth was not included in our analysis, and the poverty variable was only borrowed from the baseline, despite poverty being subject to change. The sample did not include all racial/ethnic groups and is not fully representative of the general U.S. population, limiting the generalizability of the findings. All study variables were measured at the family and individual level, with no neighborhood, community, school, or policy variables used in our analysis.

## 8. Future Research Directions

Addressing the limitations identified in this study opens avenues for future research to deepen understanding and refine conclusions. First, additional longitudinal studies with robust data collection methods will enhance causal inference regarding the relationship between socioeconomic position (SEP), race/ethnicity, and epigenetic aging. Validation of epigenetic aging measures beyond PhenoAge, particularly in diverse populations and across different contexts, is warranted to ensure broader applicability and accuracy of our observations. Incorporating additional SEP indicators, such as parental education, income, employment, wealth, and their changes over time, can provide a more comprehensive understanding of socioeconomic influences on epigenetic processes. Furthermore, exploring the impact of neighborhood, community, and policy-level variables alongside individual and family-level factors could elucidate broader social determinants of health disparities in epigenetic aging. Future research efforts should strive for more inclusive sampling strategies to encompass a wider range of racial/ethnic groups, enhancing the generalizability of findings to diverse populations within the United States. Long-term follow-up studies with strategies to mitigate attrition bias are essential for capturing dynamic changes in socioeconomic status and their implications for biological aging over the life course.

## 9. Conclusion

This study highlights the long-term impact of poverty at birth on epigenetic changes, which may vary by sex. Addressing economic disparities at birth is crucial for promoting health equity and improving the overall well-being of disadvantaged male minority youth in the long term.

## Figures and Tables

**Table 1:** shows descriptive data (n = 854).

		n	%
Race	White	467	54.68
	Black	387	45.32
Ethnicity	Non-Latino	689	80.68
	Latino	165	19.32
Household Family Structure	Unwed Household	654	76.58
	Married Household	200	23.42
Sex	Female	426	49.88
	Male	428	50.12

**Table 2.** Bivariate Correlations

	1	2	3	4	5	6	7
1 Race (Black)	1.00						
2 Ethnicity (Latino)	−0.45*	1.00					
3 Income to Poverty Ratio (Age 0)	−0.22*	−0.06*	1.00				
4 Married Household (Age 0)	−0.18*	−0.01	0.47*	1.00			
5 Maternal Education (1-4) (Age 0)	−0.09*	−0.11*	0.55*	0.37*	1.00		
6 Sex (Male)	−0.07*	−0.06*	−0.01	0.04	0.00	1.00	
7 DNAm PhenoAge (Age 15)	0.22*	−0.06*	−0.10*	−0.09*	−0.05	−0.10*	1.00

* P < 0.05

**Table 3.** Summary of Structural Equation Modeling (SEM) in the Pooled Sample

	Beta	SE	95%	CI	p
Maternal Education at Childbirth					
Ethnicity (Latino)	−0.19	0.04	−0.26	−0.12	< 0.001
Race (Black)	−0.17	0.04	−0.25	−0.10	< 0.001
Intercept	2.40	0.07	2.26	2.54	< 0.001
					
Income to Poverty Ratio at Childbirth					
Maternal Education at Childbirth	0.44	0.03	0.38	0.49	< 0.001
Married Household at Childbirth	0.30	0.03	0.24	0.36	< 0.001
Ethnicity (Latino)	−0.09	0.03	−0.15	−0.03	0.003
Race (Black)	−0.17	0.03	−0.23	−0.11	< 0.001
Intercept	0.05	0.08	−0.11	0.21	0.505
					
Married Household at Childbirth					
Ethnicity (Latino)	−0.12	0.04	−0.19	−0.04	0.002
Race (Black)	−0.23	0.04	−0.30	−0.16	< 0.001
Intercept	0.82	0.06	0.71	0.93	< 0.001
					
DNA Methylation (Age 15)					
Income to Poverty Ratio at Childbirth	−0.09	0.03	−0.16	−0.03	0.004
Sex (Male)	−0.10	0.03	−0.17	−0.04	0.002
Intercept	4.32	0.11	4.10	4.53	< 0.001

DNA Methylation measured using PhenoAge clock.

**Table 4.** Summary of Structural Equation Modeling (SEM) by Sex

	Coefficient	std. err.	[95% conf.	interval]	P>z
**Females**					
Maternal Education at Childbirth					
Ethnicity (Latino)	−0.19	0.05	−0.30	−0.08	0.001
Race (Black)	−0.19	0.05	−0.29	−0.08	0.001
Intercept	2.42	0.11	2.21	2.63	< 0.001
					
Income to Poverty Ratio at Childbirth					
Maternal Education at Childbirth	0.46	0.04	0.39	0.54	< 0.001
Married Household at Childbirth	0.28	0.04	0.20	0.36	< 0.001
Ethnicity (Latino)	−0.12	0.05	−0.21	−0.03	0.009
Race (Black)	−0.18	0.05	−0.27	−0.09	< 0.001
Intercept	0.04	0.12	−0.19	0.27	0.742
					
Married Household at Childbirth					
Ethnicity (Latino)	−0.15	0.05	−0.26	−0.05	0.005
Race (Black)	−0.25	0.05	−0.35	−0.14	< 0.001
Intercept	0.85	0.08	0.68	1.01	< 0.001
					
DNA Methylation (Age 15)					
Income to Poverty Ratio at Birth	−0.06	0.05	−0.15	0.03	0.206
Intercept	4.53	0.16	4.21	4.85	< 0.001
					
**Males**					
Maternal Education at Childbirth					
Ethnicity (Latino)	−0.20	0.05	−0.30	−0.10	< 0.001
Race (Black)	−0.17	0.05	−0.27	−0.07	0.001
Intercept	2.39	0.10	2.19	2.58	< 0.001
					
Income to Poverty Ratio at Childbirth					
Maternal Education at Childbirth	0.40	0.04	0.32	0.49	< 0.001
Married Household at Childbirth	0.33	0.04	0.25	0.42	< 0.001
Ethnicity (Latino)	−0.07	0.04	−0.16	0.01	0.095
Race (Black)	−0.18	0.04	−0.26	−0.09	< 0.001
Intercept	0.10	0.11	−0.13	0.32	0.400
					
Married Household at Childbirth					
Ethnicity (Latino)	−0.07	0.05	−0.18	0.03	0.144
Race (Black)	−0.22	0.05	−0.31	−0.12	< 0.001
Intercept	0.80	0.07	0.65	0.94	< 0.001
					
DNA Methylation (Age 15)					
Income to Poverty Ratio at Birth	−0.13	0.05	−0.22	−0.04	0.005
Intercept	3.99	0.14	3.70	4.27	< 0.001

DNA methylation was measured using PhenoAge biological clock at age 15.

## Data Availability

“FFCWS data are publicly available at the Office of Population Research data repository: https://oprdata.princeton.edu/archive/restricted/Default.aspx.”
